# ENGAGING IN CQI TO ENHANCE ADULT PROTECTIVE SERVICES INTAKE AND SCREENING: A QUALITATIVE ANALYSIS

**DOI:** 10.1093/geroni/igac059.2754

**Published:** 2022-12-20

**Authors:** Joan Davitt, Jocelyn Brown, Peiyuan Zhang, Erin Barry-Dutro, Greg Sesek

**Affiliations:** University of Maryland, School of Social Work, Baltimore, Maryland, United States; University of Maryland, Baltimore, Baltimore, Maryland, United States; University of Maryland, Baltimore, Baltimore, Maryland, United States; University of Maryland, Baltimore, Baltimore, Maryland, United States; Department of Human Services, Baltimore, Maryland, United States

## Abstract

This qualitative evaluation study was part of a larger Continuous Quality Improvement initiative implemented by the Maryland Department of Human Services Office of Adult Services to improve the Adult Protective Services (APS) screening and intake process statewide. Researchers conducted two 90-minute focus groups via Zoom with 21 intake and screening staff from 14 counties in Maryland in the summer of 2022. Focus group participants were mostly White and African American women with an average of 6.5 years working in adult services. Interviews focused on understanding challenges in APS intake/screening and strategies to overcome those challenges. Each interview was recorded and transcribed verbatim and three researchers used open and axial coding to identify themes. The key challenge expressed by all participants was the difficulty obtaining adequate information from referral sources, in order to discern whether the adult was vulnerable and if the alleged abuse/neglect/exploitation was being committed by a trusted caregiver or fiduciary as defined by state law. Inadequate referral information results in supervisors/screeners making time-consuming follow-up calls to obtain additional information or cases inappropriately screened in for investigation. This challenge was exacerbated by intake workers inexperienced in APS, understaffing, unclear policies and screening tools for APS, and community partners’ and the public’s unfamiliarity with APS services and policy. Strategies to overcome these challenges included additional staff training, public education, additional resources for APS, clearer policies, and improving the APS screening tool. Quality improvement strategies developed from this study that are relevant to APS systems nationwide will be discussed.

